# Molecular-Field-Based Three-Dimensional Similarity Studies on Quinoline-Based CNS Active Agents

**DOI:** 10.5402/2011/186943

**Published:** 2011-09-07

**Authors:** Alka Bali

**Affiliations:** University Institute of Pharmaceutical Sciences, Panjab University, Chandigarh 160014, India

## Abstract

A series of quinoline-based agents with CNS activity have been analyzed for their three-dimensional similarity with respect to a set of standard atypical antipsychotics. The method aligns the molecules based on their molecular fields represented as local extrema of electrostatic, van der Waals, and hydrophobic potentials of the molecule termed “field points.” The compounds in the series were found to demonstrate relatively lesser 3D similarity to the dibenzodiazepine derivative clozapine. Similarity values were higher with respect to extended chain compounds ketanserin, ziprasidone, and risperidone. The results obtained were found to agree with the physicochemical similarity of the compounds reported earlier.

## 1. Introduction

Molecular similarity is one of the most widely used concepts in the computer-aided approaches to molecular design. According to the “molecular similarity principle,” compounds with similar chemical structures are more likely to possess similar physicochemical and biological activities [1–6]. Despite recent examples that one cannot conclude property similarity from structural similarity in every case [7], this is still the underlying assumption of current drug design efforts which includes building QSAR models and using them for making predictions for new compounds. Some studies also indicate that structural similarity does not always imply similarity in descriptors [8].

The concept of chemical similarity does not have a formal definition, and it may vary depending upon the application for which similarity needs to be determined. Quantification of chemical similarity is based on numerical representation of a chemical structure, and similarity comparisons are done based on 2D or 3D approaches. 2D approaches involve the calculation of fingerprint-based [9] or descriptor-based similarity [2]. The former involves a simple count of shared features (common fragment substructures) as a measure of quantifying the degree of structural resemblance. In the latter case, similarity can be based on descriptors such as continuous whole molecule properties, for example, log *P*, molar refractivity, and topological indexes. Various similarity metrics exist that return a score indicating the level of similarity between molecules under comparison. Frequently used metrics are simple distance measures such as Hamming and Euclidean distance and association coefficients such as Tanimoto, Dice, and Cosine coefficients [10]. Tanimoto coefficient has been the measure of choice for fragment-based chemical similarity work, whereas euclidean distance measurement is the most popular measure for continuous data (descriptors). 

3D methods can be alignment-independent methods based on descriptors such as geometric atom pairs and their distances, valence and torsion angles, and atom triplets and alignment methods, which consider the conformational flexibility of the molecules. Field-based alignment methods [11] are based on quantum mechanical calculations, and the similarity score is related to the electron density of the molecules which involves calculation of steric fields (van der Waals surface) and electrostatic fields (derived from precalculated point charges). Examples include methods like MIMIC [12] using fixed conformations in matching process. Flexible-field-based alignment (FLASHFLOOD) [13] is preferred over conformationally rigid matching [14, 15]. Comparable activities of diverse molecules at the same molecular targets can be explained by considering the molecules' fields rather than their atomic structure because the field pattern is a far superior description of molecule's binding properties than its atomic structure. Compounds which are structurally diverse but show comparable activity have similar fields and, hence, similar binding properties so that these can bind to the same target site and elicit the same biological effect. 

We had recently reported a novel series of quinoline derivatives evaluated for their atypical antipsychotic potential [16] and further assessed for their 2D similarity (physicochemical similarity) with respect to the standard atypical antipsychotic drugs. In the present research paper, we report the 3D similarity (based on field similarity, volume similarity, and shape similarity) of this series of compounds to the standard drugs clozapine, risperidone, ziprasidone, and ketanserin based upon field-based alignment methods.

## 2. Method

Field based similarity of the compound set was assessed with respect to the standard drugs taken as reference, that is, clozapine, ketanserin, ziprasidone and risperidone using *FieldAlign2.1.1* (Cresset BioMolecular Discovery Ltd., UK). The three dimensional models of the reference drugs were generated using ChemBio3D Ultra 12.0 and energy minimization was performed with MM2 force field to minimum RMS gradient of 0.100. These reference drugs in a defined 3D conformation were then imported in sdf (MDL mol) format to *FieldAlign2.1.1*. Molecules to be aligned were imported in 2D from ChemBioDraw Ultra 12.0 as sdf (MDL mol) files. The maximum number of conformations generated for any molecule was limited to 200 in order to have a balance of the quality of alignments and calculation time. Number of high temperature dynamics for flexible rings was set at 5. Gradient cut-off for conformer minimization was 0.5. Coarseness of the sampling of conformational space was controlled by filtering duplicate conformers at rms 0.5. Standard scoring function was used based on 50% shape similarity and 50% dice volume similarity to derive overall similarity between two conformations. Further, a 3D reference template was generated taking three reference drug molecules at a time (loaded as single 2D structures ) using *FieldTemplator2.1.1 *(Cresset BioMolecular Discovery Ltd., UK) that searches for common field patterns across the explored conformational space of a set of ligands looking for commonality. The best template was selected based on their field similarity, shape similarity and overall similarity scores.

## 3. Results and Discussion

Table 1 shows the chemical structures and pharmacological activity profile of the quinoline-based compound set (**7**–**14**) taken for the 3D alignment studies. The overall alignment (similarity) scores along with the corresponding field similarity, volume similarity, and shape similarity scores of the test molecules with respect to a selection of standard drugs are shown in Tables 2, 3, and 4. The alignment scores serve as a measure of how similar the molecular fields of the two molecules are in the given alignment. 

**Figure 1:**
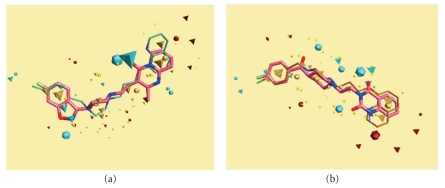
Alignment of compound **14** (thin sticks) with risperidone (a) and ketanserin (b). Risperidone and ketanserin are shown as capped sticks (pink). Tetrahedra and dodecahedra depict field points for **14** and reference compounds, respectively. Blue, maroon, yellow, and brown colors depict negative field, positive field, surface field, and hydrophobic field points.

**Figure 2:**
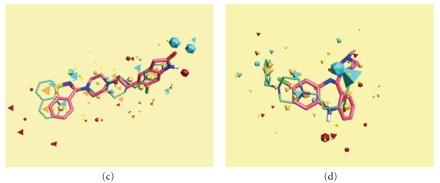
Alignment of compound **14** (thin sticks) with ziprasidone (c) and clozapine (d). Ziprasidone and clozapine are shown as capped sticks (pink). Field point depiction is the same as in Figure 1.

**Figure 3:**
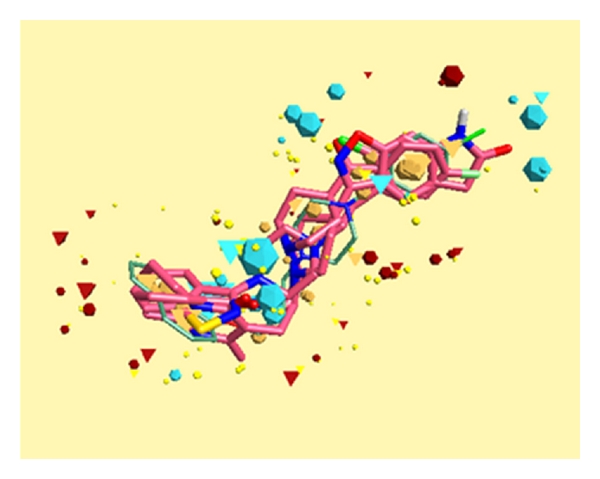
Alignment of compound **14** (thin sticks) with three compound templates generated from risperidone, ketanserin, and ziprasidone shown as capped sticks (pink). Field point depiction is the same as in Figure 1.

Figures 1–3 show the graphic display of the best alignment (highest alignment score) of the compound **14** with standard drugs along with the various field points. Compound **14 **is the lead compound which had demonstrated a potential atypical antipsychotic profile in our earlier studies. The best alignment obtained with risperidone shows the field superposition of quinoline and piperazine nitrogens in **14 **with corresponding nitrogens in the drug. The overlap of negative field points corresponding to the ether oxygen in **14 **and carbonyl oxygen in the drug is also seen as a major contributor to field similarity. The quinoline nitrogen does not, however, assume correspondence with any of the two heterocyclic nitrogens in risperidone. However, good intersection of quinoline and quinazoline nitrogens is seen in addition to the superposition of negative field points of ether moiety and carbonyl group, piperazine and piperidine nitrogens, and the corresponding halogen atoms in **14 **and ketanserin, respectively, which accounts for higher similarity of **14 **to ketanserin than to risperidone. 

The best alignment of **14 **with ziprasidone shows the overlay of field points corresponding to ether oxygen in **14 **with benzothiazole nitrogen in the drug. Further, field superposition of chlorine in **14 **with carbonyl oxygen of indolinone moiety in the drug contributes towards the overall similarity between the two. In comparison to these drugs, there is less superposition of the field points of **14 **with clozapine accounting for lower similarity scores in this case. Further, the alignment of **14 **with the three compound template generated from risperidone, ketanserin and ziprasidone does not improve the similarity scores as evident from the graphic display in Figure 3.

The overall similarity scores of the compounds were found to increase with chlorination with respect to risperidone, ziprasidone and ketanserin. However, compounds **10 **and **14 **showed nearly same similarity values with respect to clozapine. 

The 8-quinoline based compounds were found to have higher similarity score with respect to risperidone and ketanserin than the corresponding 6-quinoline derivatives. However, with respect to ziprasidone, 6-quinoline compounds have a higher similarity (except **14 **versus **12**). Further, a pattern of increase in similarity scores with respect to the selected standard drugs was noted which was different for the 8- and 6-quinoline series. The similarity for the former was in the order clozapine<risperidone<ziprasidone <ketanserin. Order for the 6-quinoline series was clozapine<risperidone<ketanserin<ziprasidone.

The field similarity with respect to ziprasidone increased with chlorination for 8-quinoline derivatives and decreased for 6-quinoline derivatives. With respect to the other drug molecules, chlorination in general, increased the field similarity of the compounds. A comparison of 6- and 8-quinoline derivatives in terms of their field similarity scores showed that 8-quinoline derivatives have a greater field similarity to ziprasidone than their corresponding 6-quinoline analogs. However, with respect to clozapine, scores were not significantly different.

Shape similarity with respect to risperidone and ziprasidone (except 8-quinolyl benzoyl derivative **7**) showed an increase with chlorination. No particular pattern was seen on chlorination with respect to ketanserin. A comparison between 6- and 8-quinolyl derivatives showed that, for nonchlorinated compounds, the latter showed higher shape similarity to ketanserin and clozapine whereas, for chlorinated analogs, 6-quinoline derivatives showed higher scores. 

Further, as expected, the volume similarity values were not significantly changed on chlorination or in the comparison of the 6- and 8-quinolyl derivatives.

The average similarity scores for the compounds were the highest with respect to ketanserin followed by ziprasidone and risperidone. Interestingly, the physicochemical similarity for the compounds reported earlier was the lowest with respect to ketanserin for the same drug group (90.64%, 85.5%, and 76.7% with respect to ziprasidone, risperidone and ketanserin, resp.). In line with the results from our previous computational studies, wherein the physicochemical similarity was the lowest with respect to the more compact dibenzodiazepine derivative clozapine (56.85%), the 3D similarity values were also found to be the lowest with respect to clozapine as well as the conventional neuroleptic chlorpromazine. 

An analysis of the breakup of the overall alignment scores shows that the contribution of the shape and volume similarity was significantly greater than the corresponding field similarity values in all the cases. As can be seen from the tables, the shape similarity and volume similarity is more than 0.7 for all the drug examples (except clozapine) whereas their field similarity scores range from 0.54 to 0.58.

## 4. Conclusions

In the present work, a set of quinolyloxypropyl piperazine derivatives have been analyzed for their three-dimensional similarity to a selection of atypical antipsychotic drugs, and these demonstrate higher similarity with respect to the extended chain structures such as risperidone, ziprasidone, and ketanserin and lesser similarity to the prototypic agent clozapine. Further, specific patterns were observed for the change in similarity scores with change in chemical structure. An introspection of the field alignments obtained for the molecules (especially, the lead compound from our studies **14**) with respect to standard drugs suggests a good correspondence of quinoline nitrogen, piperazine system, ether oxygen, and chlorine atom with the corresponding groupings in the standard drug molecules. This highlights the importance of these structural features as a part of the chromophoric system involved in the pharmacological activity of this class of compounds. Further development of this compound series can be carried out by appropriate modifications whilst retaining these salient features. Hence, the information generated from the molecular field analysis of this compound series can be used as a valuable tool for designing novel analogues by interpretation of their pharmacological activity in terms of their field pattern.

##  Conflict of Interests

The author declares no conflict of interests.

## Figures and Tables

**Table 1:** Chemical structures and results in pharmacological testing [16].

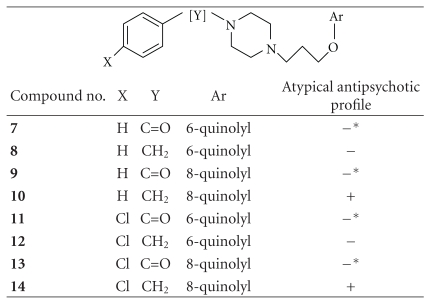

*Show blockade only in apomorphine-induced stereotypy assay indicative of potential to cause extrapyramidal symptoms.

**Table 2:** Similarity of test compounds with respect to risperidone and ketanserin.

Compd. no.	Similarity		Field similarity		Field score		Vol. Similarity		Volume score		Shape similarity		Shape score	
	Ris.	Ket.	Ris.	Ket.	Ris.	Ket.	Ris.	Ket.	Ris.	Ket.	Ris.	Ket.	Ris.	Ket.
**7**	0.620	0.672	0.483	0.539	−48.519	−52.436	0.806	0.787	204.854	193.544	0.756	0.804	192.146	197.828
**8**	0.619	0.648	0.541	0.480	−50.853	−46.808	0.709	0.784	201.876	191.679	0.697	0.815	175.958	199.110
**9**	0.659	0.686	0.552	0.576	−55.523	−57.483	0.783	0.802	198.906	197.155	0.766	0.795	193.085	193.834
**10**	0.610	0.683	0.525	0.565	−49.877	−55.710	0.748	0.772	189.082	188.609	0.696	0.801	175.690	195.756
**11**	0.661	0.689	0.542	0.594	−53.226	−59.520	0.740	0.799	194.220	203.325	0.781	0.783	205.048	199.232
**12**	0.626	0.659	0.512	0.501	−48.374	−49.907	0.770	0.803	200.828	202.951	0.740	0.818	193.227	206.720
**13**	0.676	0.721	0.581	0.635	−56.250	−64.061	0.824	0.820	216.246	208.667	0.772	0.808	202.659	205.442
**14**	0.658	0.716	0.549	0.610	−53.264	−58.534	0.745	0.802	194.555	202.734	0.768	0.823	200.627	208.108
**Average**	**0.641**	**0.684**	**0.536**	**0.563**	−**51.986**	−**55.557**	**0.766**	**0.796**	**200.070**	**198.583**	** 0.747**	**0.806**	**192.305**	**200.754**

**Table 3:** Similarity of test compounds with respect to ziprasidone and clozapine.

Compd. no.	Similarity		Field similarity		Field score		Vol. similarity		Volume score		Shape similarity		Shape score	
	Zip.	Clz.	Zip.	Clz.	Zip.	Clz.	Zip.	Clz.	Zip.	Clz.	Zip.	Clz.	Zip.	Clz.
**7**	0.696	0.566	0.639	0.540	−66.121	−50.871	0.798	0.620	201.876	141.337	0.752	0.592	190.325	134.964
**8**	0.651	0.567	0.564	0.482	−58.038	−41.151	0.741	0.649	186.210	147.078	0.738	0.652	185.787	147.697
**9**	0.652	0.558	0.557	0.521	−58.139	−47.685	0.772	0.589	195.325	134.285	0.747	0.595	187.604	134.470
**10**	0.632	0.579	0.575	0.484	−59.279	−43.358	0.708	0.586	178.021	132.880	0.689	0.674	173.209	152.661
**11**	0.703	0.583	0.603	0.553	−59.824	−49.015	0.730	0.624	190.885	147.619	0.802	0.622	209.751	147.293
**12**	0.603	0.590	0.457	0.556	−45.789	−48.918	0.778	0.569	202.276	133.575	0.749	0.625	194.708	146.938
**13**	0.675	0.571	0.599	0.556	−62.140	−52.714	0.786	0.615	205.549	145.517	0.711	0.585	185.959	138.252
**14**	0.675	0.573	0.606	0.539	−63.004	−49.314	0.730	0.654	189.892	153.728	0.744	0.606	193.411	142.511
**Average**	**0.661**	**0.573**	**0.575**	**0.529**	−**59.042**	−**47.878**	**0.755**	**0.613**	**193.754**	**144.002**	** 0.742**	**0.619**	**190.094**	**143.098**

**Table 4:** Similarity of test compounds with respect to chlorpromazine and template.

Compd. no.	Similarity		Field similarity		Field score		Vol. similarity		Volume score		Shape similarity		Shape score	
	CPZ	Temp	CPZ	Temp	CPZ	Temp	CPZ	Temp	CPZ	Temp	CPZ	Temp	CPZ	Temp
**7**	0.613	0.603	0.535	0.466	−48.258	−47.987	0.628	0.620	141.826	141.337	0.690	0.740	164.092	185.972
**8**	0.565	0.579	0.487	0.469	−41.443	−46.261	0.641	0.649	147.210	147.078	0.643	0.689	151.980	172.277
**9**	0.557	0.575	0.532	0.451	−49.327	−45.631	0.572	0.589	135.325	134.285	0.583	0.699	137.385	174.194
**10**	0.561	0.576	0.517	0.425	−46.640	−41.811	0.588	0.586	138.021	132.880	0.606	0.728	143.205	181.874
**11**	0.543	0.596	0.493	0.456	−46.066	−44.811	0.630	0.624	141.855	147.619	0.592	0.736	145.862	191.305
**12**	0.581	0.582	0.500	0.436	−43.990	−44.166	0.578	0.569	136.276	133.575	0.662	0.728	162.107	188.021
**13**	0.599	0.635	0.520	0.543	−48.090	−54.255	0.616	0.615	145.549	145.517	0.679	0.726	167.193	188.809
**14**	0.591	0.618	0.538	0.489	−51.045	−49.919	0.630	0.654	142.092	153.728	0.644	0.748	157.594	193.364
**Average**	**0.576**	**0.596**	**0.515**	**0.467**	**−46.857**	**−46.855**	**0.610**	**0.613**	**141.019**	**142.002**	**0.637**	**0.724**	**153.677**	**184.477**
